# Supplementary material for: Prevalence and associating factors for iatrogenic gliosis-like changes in surgically treated intracranial meningioma patients—A retrospective study of 255 meningioma patients
Source: PLoS One. 2025 Dec 30;20(12):e0339857. doi: 10.1371/journal.pone.0339857 (PMC12752940; doi:10.1371/journal.pone.0339857)
Supplement: S1 Table — IGC = Iatrogenic gliosis-like change, IM = Intracranial meningioma, IR = Interquartile range, KPS = Karnofsky performance status, MRI = Magnetic resonance imaging, SD = Standard deviation, WHO = World Health Organization. (DOCX) [file pone.0339857.s001.docx]

Supplementary Table 1. Characteristics of patients with postoperative seizures

|  | Overall (n=255) | New-onset seizures (n=8) | Persisting seizures (n=9) |
| --- | --- | --- | --- |
| Age in years, median (IR) | 54.0 (45.5-63.0) | 52.5 (48.5-57.3) | 53.0 (48.0-59.0) |
| Age group, n (%)  Young adult (18-39)  Middle aged adult (40-64)  Older adult (65+) | 28 (11.0%)  173 (67.8%)  54 (21.2%) | 8 | 1  7  1 |
| Sex, n (%)  Women  Men | 200 (78.4%)  55 (21.6%) | 6  2 | 5  4 |
| IM location, n (%)  Convexity  Skull base  Parasagittal  Falx  Intraventricular/Tentorial | 95 (37.3%)  86 (33.7%)  33 (12.9%)  32 (12.5%)  9 (3.5%) | 3  2  2  1 | 6  1  1  1 |
| WHO Grade, n (%)  I  II | 237 (92.9%)  18 (7.1%) | 7  1 | 7  2 |
| IM volume in cm^3^, median (IR) | 3.0 (1.2-9.1) | 2.9 (1.9-32.0) | 3.7 (3.0-22.2) |
| Preoperative IM T2-intensity  Isointense  Hyperintense  Hypointense | 162 (63.5%)  82 (32.2%)  11 (4.3%) | 4  4 | 6  3 |
| Postoperative IGC present, n (%)  Yes  No | 133 (52.2%)  122 (47.8%) | 7  1 ( | 6  3 |
| Postoperative IGC volume, median (IR) | 0.1 (0.0-0.8) | 1.6 (0.1-7.8) | 0.2 (0.0-1.0) |
| IGC location, n (%)  Frontal  Temporal  Parietal  Occipital | 92 (69.2%)  16 (12.0%)  16 (12.0%)  9 (6.8%) | 7 (7.6%) | 5 (5.4%)  1 (6.3%) |

IGC = Iatrogenic gliosis-like change, IM = Intracranial meningioma, IR = Interquartile range, KPS = Karnofsky performance status, MRI = Magnetic resonance imaging, SD = Standard deviation, WHO = World Health Organization
