# Supplementary material for: Prevalence and associating factors for iatrogenic gliosis-like changes in surgically treated intracranial meningioma patients—A retrospective study of 255 meningioma patients
Source: PLoS One. 2025 Dec 30;20(12):e0339857. doi: 10.1371/journal.pone.0339857 (PMC12752940; doi:10.1371/journal.pone.0339857)
Supplement: Attachment — Submitted filename: Response to Reviewers.docx [file pone.0339857.s002.docx]

**Department of Neurosurgery**

Submission ID: PONE-D-25-39472

MS title: ” Prevalence and associating factors for iatrogenic gliosis-like changes in surgically treated intracranial meningioma patients – A retrospective study of 255 meningioma patients”

Dear Dr Ryota Tamura,

We would like to express our gratitude for your consideration and the valuable feedback provided by the referees regarding our manuscript. We are grateful for the opportunity to submit a revised version. The reviewers have brought up important aspects that were all taken into account when revising the manuscript. In other words, we have made every effort to address all the comments and questions. Below, please find our detailed responses to the editor and reviewers. Thank you very much.

On behalf of all authors,

**Editor**

1. Please ensure that your manuscript meets PLOS ONE's style requirements, including those for file naming. The PLOS ONE style templates can be found at

Thank you for highlighting the PLOS ONE style requirements, including the guidelines for file naming. We have revised and reformatted the manuscript to ensure full compliance with the PLOS ONE formatting standards and sample templates.

2.

a) If there are ethical or legal restrictions on sharing a de-identified data set, please explain them in detail (e.g., data contain potentially identifying or sensitive patient information, data are owned by a third-party organization, etc.) and who has imposed them (e.g., a Research Ethics Committee or Institutional Review Board, etc.). Please also provide contact information for a data access committee, ethics committee, or other institutional body to which data requests may be sent.

This study was approved by the Ethics Board of Helsinki University Hospital (HUH). Under Finnish legislation, register-based studies do not require individual patient consent. Due to legal data-transfer restrictions imposed by the European Union, the de-identified dataset used in this study cannot be shared directly. Requests for access to the original data may be submitted to the Finnish Health and Social Data Permit Authority (Findata): <http://findata.fi/en/>.

3. If the reviewer comments include a recommendation to cite specific previously published works, please review and evaluate these publications to determine whether they are relevant and should be cited. There is no requirement to cite these works unless the editor has indicated otherwise.

Thank you for the clarification. The reviewers did not request the citation of any specific previously published works.

4. Please review your reference list to ensure that it is complete and correct. If you have cited papers that have been retracted, please include the rationale for doing so in the manuscript text, or remove these references and replace them with relevant current references. Any changes to the reference list should be mentioned in the rebuttal letter that accompanies your revised manuscript. If you need to cite a retracted article, indicate the article’s retracted status in the References list and also include a citation and full reference for the retraction notice.

Thank you for the guidance. We have reviewed the entire reference list and confirmed that all entries are complete and accurate. None of the cited works have been retracted. No changes to the reference list were required.

**Reviewer #1**

Dear Author,

I read and reviewed your article titled "Prevalence and Associated Factors for Iatrogenic Gliosis-Like Changes in Surgically Treated Intracranial Meningioma Patients – A Retrospective Study of 255 Meningioma Patients" with interest. This is a study that will guide the literature. Thank you.

We sincerely thank the Reviewer for the delightful feedback.

**Reviewer #2**

Dear Author,

I had the opportunity to review your study titled "Prevalence and Associated Factors for Iatrogenic Gliosis-Like Changes in Surgically Treated Intracranial Meningioma Patients – A Retrospective Study of 255 Meningioma Patients."
I believe your results, which are consistent with the literature, will be beneficial in the scientific arena.

Thank you.

We are grateful to the Reviewer for the time invested in reviewing our manuscript.

**Reviewer #3:**

This is a follow-up manuscript from a single center retrospective cohort of surgically treated patients with meningioma that aimed to measure the odds of developing IGC as diagnosed with follow-up MRI. Authors conclude that the presence of IGC was associated with GTR of large and non-convexity intracranial meningiomas. The article is well presented and easy to follow.
Minor areas of opportunity are:

1: In table 1 and 2 please consider changing WHO Grade from roman numbers to arabic. (1 instead of I, and 2 instead of II).

We thank the Reviewer for this relevant and appropriate comment. Accordingly, we have made the following changes to the manuscript:

Table 1 and Table 2: WHO grading changed to Arabic numerals.

2: Self-citation is not encouraged, see references 9 and 11.

Thank you very much for this excellent remark. We fully agree that self-citations should be avoided. In this case, however, we hope that the citations are appropriate, as the cited findings and facts are available only in our earlier publications.

3: As this was an exploratory study, hypothesis could be obviated; nevertheless, authors wrote two hypothesis, these hypothesis are usually used to calculate the sample size (which was not done) considering the direction and magnitude of the null hypothesis. Reconsider and follow proper research methods

We again thank the reviewer for the professional comments. We have made the following changes to the manuscript:

Under abstract, Manuscript file page 2 line 43-44: ~~We hypothesized that ICGs are infrequent but impact outcomes.~~

Under introduction, Manuscript file page 3 line 93-95: ~~We hypothesized that persistent IGCs would occur infrequently. Furthermore, we hypothesized that IGCs associate with impaired surgical outcomes.~~  🡪 Our aim was to estimate the prevalence of persistent IGCs and to explore whether the presence of persistent IGCs is associated with surgical outcome.

Under discussion, Manuscript file page 10 line 262: ~~Contrary to our hypothesis,~~

4: Was RANO, QLQ-BN20 considered at any time?

We applied RANO principles when assessing postoperative imaging. All meningioma measurements were performed on T1-weighted MRI, and in this cohort every tumor was completely resected with no recurrences observed. While a full RANO-based reporting framework is generally applied in clinical trials, our study was observational and not trial-based, so comprehensive RANO reporting was not performed.

We agree that QLQ-BN20 data would add valuable insight. Unfortunately, quality-of-life measures are not routinely collected at our institution, and these data were therefore unavailable for this study. We have added the following statement to the limitations section:

Under limitations, Manuscipr file page 12 line 313: “Quality of life data was also not available”

5: It is easier to decribe the P value as <0.01 rather than the negative exponential (Table 2); and the 95%CI is almost never the same value as the OR (i.e., OR 1.1; 95% CI, 1.1, 1.2). OR for non-convexity vs convexity should also be included for one of the conclusions is precisely this. This last comment should is also to be considered for GTR vs non-GTR.

We thank the Reviewer for the comments that surely improve the manuscript. We absolutely agree that reporting p-values without negative exponents improves clarity, and therefore we have updated Table 2 accordingly. We also agree that a convexity versus non-convexity analysis is informative. Unfortuntaly, as all included meningiomas in this study were completely resected (GTR), a GTR versus non-GTR comparison was not possible.

The following changes were made:

**Table 2:** P-values previously reported with negative exponents were updated to standard format (e.g., <0.001).

**Table 2:** A new row for **“Convexity”** was added, including the univariate p-value and odds ratio. This variable was not included in the multivariate analysis due to multicollinearity with IM location.

6: Large volume cut-off value should be included or the phrase large should change (suggestion: presence of IGC was associated with GTR (place OR, 95% CI, and P values), volume (place OR, 95%CI, and P values), and non-convexity (place OR, 95%CI, and P value).

We thank the Reviewer for the comment. The analysis was conducted using continuous volume increases, and we observed that each 1 cm³ increase in volume was associated with an increased risk of IGCs. To clarify this point, we have made the following changes:

Under results, manuscript page 8 line 215: “increasing IM volume (OR 1.1 **per 1 cm^3^** increase, p<.001)

Under results manuscript page 9 line 250-251: ~~This is likely related to larger IM volumes and a higher frequency of WHO grade 2 IMs in these patients (Table 2).~~

Congratulations on your line of research

We sincerely thank the Reviewer for thorough and insightful review, which has greatly helped us to improve the clarity and quality of our manuscript.

**Reviewer #4**

I have only 1 question:

Patients who underwent previous radiation therapy were excluded. However in 18 patients a WHO grade 2 meningioma was diagnosed. Did they undergo radiation treatment only after the first MRI at least one year later?

We thank the Reviewer for the valid question. In our study, we had 18 patients with grade 2 meningiomas, and all surgeries were complete resections. The operations took place between 2005 and 2018. These grade 2 meningiomas were on the less aggressive side of atypical meningiomas, so no patient received radiation therapy right after surgery. During follow-up after the study period, 3 patients needed radiation therapy because of radiological recurrence. The first patient had surgery in 2010 and received radiation in 2015. The second patient had surgery in 2016 and received radiation in 2019. The third patient had surgery in 2018 and received radiation in 2023.

And one textual remark: On p3, line 87/88, this sentence may improve when the authors write: ..., we assessed the occurrence of persistent FLAIR hyper intensity changes in IM patients without preoperative PTBE, who underwent gross total resection (GTR).

We thank the Reviewer for the good suggestion. We revised the manuscript as the Reviewer proposed:

Under introduction, Manuscript file page 3 line 86-88: “…,we assessed the occurrence of persistent FLAIR hyperintensity changes in IM patients who underwent gross total resection (GTR) without preoperative PTBE” 🡪 “…,we assessed the occurrence of persistent FLAIR hyperintensity changes in IM patients without preoperative PTBE who underwent gross total resection (GTR).”
