# Supplementary material for: Prevalence and associating factors for iatrogenic gliosis-like changes in surgically treated intracranial meningioma patients—A retrospective study of 255 meningioma patients
Source: PLoS One. 2025 Dec 30;20(12):e0339857. doi: 10.1371/journal.pone.0339857 (PMC12752940; doi:10.1371/journal.pone.0339857)
Supplement: Attachment — Submitted filename: Response_to_Reviewers_auresp_2.docx [file pone.0339857.s003.docx]

**Department of Neurosurgery**

Submission ID: PONE-D-25-39472R1

MS title: ” Prevalence and associating factors for iatrogenic gliosis-like changes in surgically treated intracranial meningioma patients – A retrospective study of 255 meningioma patients”

Dear Dr Ryota Tamura,

We appreciate the reviewer’s thoughtful feedback and welcome the opportunity to provide a revised manuscript. The comments were carefully considered and have been fully addressed in the updated version. Our response is provided below. Thank you for your constructive input.

On behalf of all authors,

**Reviewer #3**

All comments were addressed; with these minor changes the article is suitable for publication in this highly recognized Journal.

Thank you for your continued review. We appreciate your initial comments and are pleased that our revisions have fully addressed them. We are grateful for your positive assessment of the manuscript.

**Reviewer #4**

I would favour adding one sentence about the radiation, just to avoid that readers think these iatrogenic changes may be the result of radiation. Otherwise, great paper.

Thank you for the helpful suggestion. We have added a clarifying sentence noting that none of the grade 2 meningiomas received radiation therapy before or during the follow-up period, thereby excluding radiation-related imaging effects. We appreciate your positive evaluation

Under Results manuscript page 8 line 212-214 added: “None of the WHO grade 2 meningiomas, nor any other meningiomas in the cohort, received radiation before or during the study’s observation period, ensuring that no imaging changes could be attributed to irradiation.”
